# The Impact of Plasma Surface Treatments on the Mechanical Properties and Magnetic Performance of FDM-Printed NdFeB/PA12 Magnets

**DOI:** 10.3390/ma17102275

**Published:** 2024-05-11

**Authors:** Ana Damnjanović, Gregor Primc, Rok Zaplotnik, Miran Mozetič, Nataša Kovačević

**Affiliations:** 1 Kolektor Mobility d.o.o., SI-5280 Idrija, Slovenia; natasa.kovacevic@kolektor.com; 2 Jožef Stefan International Postgraduate School, SI-1000 Ljubljana, Slovenia; 3 Jozef Stefan Institute, Jamova cesta 39, SI-1000 Ljubljana, Slovenia; gregor.primc@ijs.si (G.P.); rok.zaplotnik@ijs.si (R.Z.); miran.mozetic@ijs.si (M.M.)

**Keywords:** additive manufacturing, Nd–Fe–B powder, plasma surface treatment, polymer-bonded magnets, permanent magnets, FDM, adhesion

## Abstract

This study presents a novel approach for improving the interfacial adhesion between Nd–Fe–B spherical magnetic powders and polyamide 12 (PA12) in polymer-bonded magnets using plasma treatments. By applying radio frequency plasma to the magnetic powder and low-pressure microwave plasma to PA12, we achieved a notable enhancement in the mechanical and environmental stability of fused deposition modeling (FDM)-printed Nd-Fe-B/PA12 magnets. The densities of the FDM-printed materials ranged from 92% to 94% of their theoretical values, with magnetic remanence (B_r_) ranging from 85% to 89% of the theoretical values across all batches. The dual plasma-treated batch demonstrated an optimal mechanical profile with an elastic modulus of 578 MPa and the highest ductility at 21%, along with a tensile strength range of 6 to 7 MPa across all batches. Flexural testing indicated that this batch also achieved the highest flexural strength of 15 MPa with a strain of 5%. Environmental stability assessments confirmed that applied plasma treatments did not compromise resistance to corrosion, evidenced by negligible flux loss in both hygrothermal and bulk corrosion tests. These results highlight plasma treatment’s potential to enhance mechanical strength, magnetic performance, and environmental stability.

## 1. Introduction

Permanent magnets, especially those based on rare earth materials, are crucial in modern high-technology applications and their integration into electronics and renewable technologies has become increasingly widespread in daily life [1]. As the demand for such materials increases, the sustainable use of rare earth elements, which are recognized as critical raw materials, is essential [2].

Among the various types of permanent magnets, neodymium iron boron (Nd–Fe–B) magnets are the most widely used owing to their superior magnetic properties. Although sintered Nd–Fe–B magnets are known for their exceptional strength, they are highly susceptible to corrosion [3]. To mitigate this, magnetic powders are often combined with polymer binders to create injection-molded or compression-molded magnets. Polymer-bonded magnets (PBMs) benefit from the polymer binder encapsulating the magnetic particles, thus protecting them from corrosion and oxidation [4]. Additionally, the rise of additive manufacturing (AM), the cornerstone of Industry 4.0, is rapidly advancing the field of permanent magnet production. Unlike traditional formative manufacturing techniques such as injection or compression molding, AM offers greater design flexibility, builds material up rather than removing it, and results in less material consumption, which is a significant advantage when working with costly materials such as rare-earth-based magnetic powders. AM technologies offer various methods for producing magnets, each with its advantages and drawbacks. Stereolithography-produced Nd–Fe–B magnets with high filler content can achieve impressive magnetic properties [5]. Powder bed fusion excels in complex geometries and mechanical properties but requires careful control of process parameters to avoid defects [6,7,8]. While binder jetting enables near-net shapes, it must overcome the issues of porosity and finish quality [9]. Fused deposition modeling (FDM) is prominent for using thermoplastic filaments with magnetic fillers but faces its own set of challenges in terms of material properties and printing consistency. The challenge lies in the production of filaments with appropriate properties for consistent printing [10]. Various polymers have been used to bond Nd–Fe–B powders, including polyamide 12 (PA12) [11], thermoplastic polyurethane [12], polyphenylene sulfide [13], and polyether ether ketone [14,15], each selected based on the intended application of the final product. Big area additive manufacturing (BAAM) bypasses the intricacies of filament production by utilizing pre-compounded materials in pellet form, which are appropriate for fabricating large objects, although this comes with the trade-offs of lower resolutions and increased costs [16,17]. BAAM-printed NdFeB/PA12 magnets have demonstrated superior magnetic properties compared with those produced by injection molding. Additionally, extrusion from slurries, such as thermosetting epoxy [18] or photopolymer resins [19], presents another avenue for PBM production, combining 3D printing with UV curing for enhanced results. It is worth noting that FDM, as well as BAAM, belongs to the material extrusion family of additive manufacturing processes, as defined by ISO/ASTM 52900 [20].

However, the efficiency and durability of PBMs significantly depend on the integrity of the composite material. In Nd–Fe–B PBMs, the potential causes of poor adhesion at the interface between the magnetic particles and polymer matrix, such as oxide layer formation on the filler during processing in air or crosslinking of the polymer, pose a significant challenge, undermining the mechanical strength and overall performance of the final product [21]. This issue is even more pronounced in additive manufacturing, where the interfacial adhesion between polymer and metal is notably weaker than within the pure polymer itself [22], further compromising the mechanical strength and overall performance of the final product. Therefore, addressing the reduction in interfacial adhesion is critical for enhancing the efficiency and extending the longevity of Nd–Fe–B PBMs, ultimately broadening their range of applications.

Strategies employing adhesion promoters, such as silanes [23,24,25] and organo-titanates [26], have been utilized to improve adhesion. Additionally, plasma technology, known for its ability to modify or enhance surface properties, such as corrosion resistance and electrical, thermal, or mechanical characteristics, is gaining popularity. It can also influence factors such as surface tension, thereby affecting adhesion and porosity [27]. Plasma treatments have been applied to diverse materials, enhancing wetting properties in various polymers, improving the adhesion of plasma-deposited coatings, reducing friction [28], upgrading biomaterials [29], and strengthening the bond between thermoplastic polymers and metal surfaces [30]. Plasma treatment is particularly economical and environmentally friendly as it requires no water or chemicals, unlike other surface-modification methods.

While plasma treatment of macroscopic samples is a mature technology [31], the adequately uniform treatment of large quantities of powder materials in a reasonable time still represents both scientific and technological challenges [27], which arise from the huge surface-to-mass ratio of the microscopic powder. The polymer powder dropping through plasma will quickly melt upon interaction with plasma species and form aggregates, which are likely to stick to the surfaces facing plasma [32]. Powder in a dish will be unevenly treated: the topmost particles will melt and will be over-treated, while those deep in the container will not be affected at all. Stirring polymer powder in a dish would help uniform treatment but the flux of plasma species should be maintained at a relatively low level to prevent overtreatment of uppermost polymer particles. The magnetic particles, on the other hand, will not melt upon treatment with a gaseous plasma sustained by a discharge of reasonable power density, so they can be treated simply by dropping through a plasma with an appropriate density of charged particles.

The aim of this study is to investigate the impact of an environmentally friendly dual-plasma treatment process on enhancing the interfacial bonding between Nd–Fe–B magnetic fillers and PA12 polymers in polymer-bonded magnets. This innovative approach, which involves modifying the surface of the Nd–Fe–B magnetic fillers with radio-frequency (RF) plasma and/or treating the PA12 polymer with low-pressure microwave plasma (MW), represents a novel contribution to the field of magnet manufacturing. By applying plasma treatment to feedstock materials, we introduce a unique method that has not been extensively explored in previous research. Plasma treatments successfully modify the surface of polyamide 12, where low-pressure microwave plasma makes PA12 wettable and often nanostructured. However, prolonged treatment hinders its melting for laser sintering [33]. Shorter treatments improved hydrophilicity without affecting meltability; adjusting gas mixtures optimized surface properties, indicating that plasma exposure and gas composition are key to tailoring PA12 surfaces [34]. We assess the effectiveness of this approach by evaluating the mechanical and magnetic properties of FDM-printed specimens, including flexural and tensile strength. Furthermore, we conduct comprehensive corrosion tests, including immersion in water at 85 °C for 1000 h and bulk corrosion testing at 120 °C for 500 h. We hypothesize that this innovative approach will not only improve the interfacial bonding between the magnetic filler and polymer matrix but also bolster the mechanical strength of the FDM-printed specimens, without detracting from their magnetic properties.

## 2. Materials and Methods

### 2.1. Materials

To fabricate filaments for FDM, we used a spherical magnetic powder based on an Nd–Pr–Fe–Co–Ti–Zr–B alloy, commercially known as MQP S, supplied by Magnequench (Singapore). The as-received magnetic powder had the following magnetic characteristics: residual remanence (B_r_) between 730–760 mT, coercivity (H_ci_) spanning from 670 to 750 kA/m, and a maximal energy product (BH_max_) fluctuating within 80–92 kJ/m^3^ [35]. Polyamide 12, which is available under the commercial label Vestosint (Evonik, Pandino, Italy), was selected as the polymer binder. As per the manufacturer’s datasheet, PA12 has a melting point of 176 °C and an approximate bulk density of 440 g/dm^3^ [36]. During the fabrication of the filament for our second batch, organo-titanate based on titanium triisostearoylisopropoxide (CAPOW^®^KR^®^TTS/H, Ken-React^®^, Bayonne, NJ, USA) was integrated as a coupling agent because of the poor adhesion between the inorganic filler and the organic polymer matrix.

### 2.2. Plasma Treatment of Magnetic Filler and Polymer Binder

The polymer powder was treated in a low-pressure microwave plasma (MW) reactor equipped with a mixing pot. The plasma was sustained using a magnetron coupled to a waveguide with a vacuum-tight window and was concentrated just beneath the window, as shown elsewhere [37]. The small penetration depth of microwaves in the electrically conductive plasma prevented the expansion of dense plasma into the entire reactor [38], so the polymer powder was treated with the diffusing plasma. The microwave source operated at the standard frequency of 2.45 GHz. The chamber was pumped with a two-stage rotary pump (Leybold, Export, PA, USA) with an ultimate pressure below 1 Pa. The pressure was measured using a Pirani vacuum gauge (Pfeiffer Vacuum, Asslar, Germany). About 300 g of PA12 powder was added to the mixing pot equipped with a stirring device, which was rotating at 20 RPM. After evacuating the plasma reactor to the ultimate pressure of about 1 Pa, oxygen was leaked via a mass flow controller at 170 sccm to reach a working pressure of 70 Pa. Plasma was sustained at the power of 500 W and the polymer powder was treated for 30 min.

**Figure 1:**
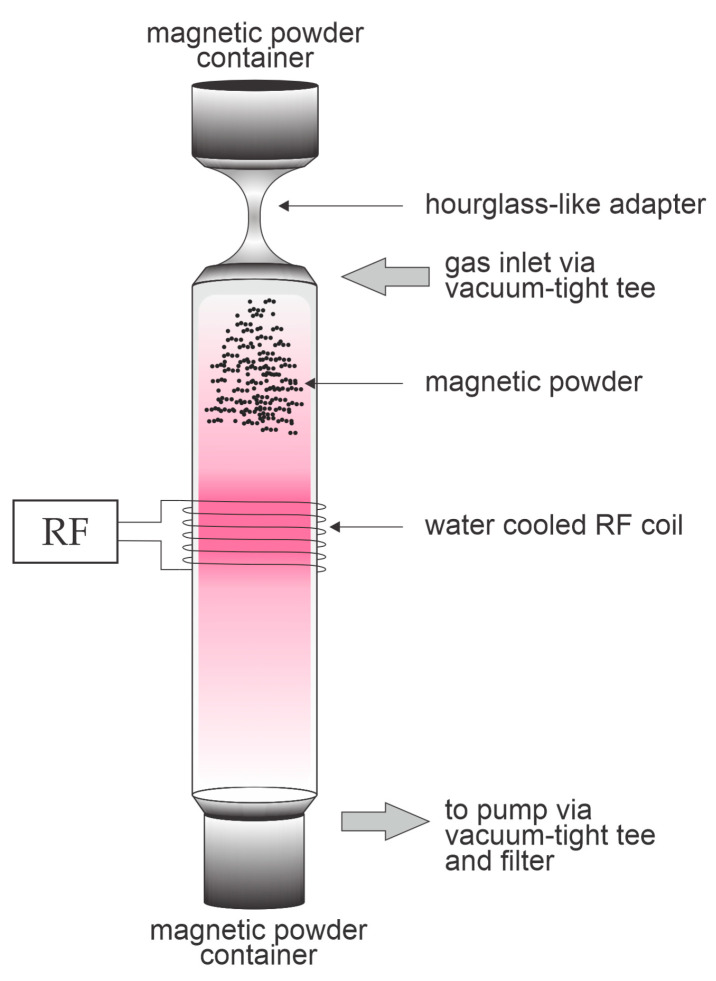
A schematic representation of a low-pressure radio frequency fall-through plasma system for the treatment of magnetic powder.

An experimental system was set up to treat magnetic powder while free-falling through non-equilibrium gaseous plasma, as shown in Figure 1. A vertical 35-cm tube with an outside diameter of 40 mm made of borosilicate glass was mounted between two vacuum flanges. A six-turn coil was wrapped around the tube and connected to a radiofrequency matching network and generator operating at the industrial frequency of 13.56 MHz (Advanced Energy, Fort Collins, CO, USA). The reactor was pumped with a two-stage rotary vacuum pump capable of achieving an ultimate pressure of 0.1 Pa (Trivac D16B, Leybold, Export, PA, USA). Oxygen was leaked via a precise needle valve and the pressure was monitored using an absolute capacitive pressure gauge (MKS Baratron, Andover, MA, USA). Both the upper and bottom vacuum flanges were connected to two powder-storage containers of volumes of about 0.4 L. Magnetic powder was then poured into a container and attached to the reactor. On one side of the container, fitted via a KF40 flange, an hourglass-like adapter was mounted to slow down the fall of magnetic powder through plasma (length 8 cm with 4.5 mm inner narrowing that is 2 cm long). The adapter dimensions can be distinguished from Figure 1. The system was evacuated to roughly 3–5 Pa. Oxygen was introduced via a needle valve to obtain the operating pressure of 35 Pa and then the plasma generator was turned on. The plasma in the H-mode was sustained in the middle of the tube. The forward and reflected powers were 500 and 20 W, respectively. The reactor was turned upside-down, so the magnetic powder fell through the powerful plasma due to gravitation. The residence time of magnetic powder in plasma was only 0.25 s. Finally, the treated powder was collected from the bottom container.

### 2.3. Filament Extrusion

We used the process described in a previous study for filament extrusion [11]. To enhance the speed and printing precision, we used MQP S as the magnetic filler. The spherical morphology of the MQP S particles was found to be advantageous, enhancing printability and resulting in filaments that were easier to print than those made with melt-spun magnetic powder. Additionally, according to the aforementioned findings, samples printed with MQP S exhibited higher density, reduced porosity, and improved corrosion resistance, demonstrating superior environmental stability with less flux loss during corrosion testing. Four distinct batches of filaments were manufactured, each with a filler loading of 93 wt.% and a polymer loading of 7 wt.%, which are presented in Table 1. The first batch, denoted as MQP S/PA12, incorporated MQP S filler bonded with PA12 without the use of coupling agents or plasma treatments. This batch served as a reference for evaluating the effects of adding a coupling agent or plasma surface treatments on the mechanical and magnetic properties of FDM-printed samples. The second batch incorporated MQP S filler bonded with PA12, with an organo-titanate as the coupling agent, and was designated as MQP S/PA12 CA. The addition of organo-titanate aimed to establish a molecular bridge at the interface between the two substrates, enhancing the bond strength and compatibility between the magnetic powder and polymer matrix [39]. The third batch resembled the first in terms of fillers and the absence of a coupling agent, yet PA12 underwent plasma treatment; this batch was labeled MQP S/PA12*. The final (fourth) batch mirrored the third batch in terms of coupling agent absence but both MQP S and PA12 underwent plasma treatment, resulting in its designation as MQP S*/PA12*. A summary of the batches is presented in Table 1.

### 2.4. Fused Deposition Modeling of Permanent Magnets

**Figure 2:**
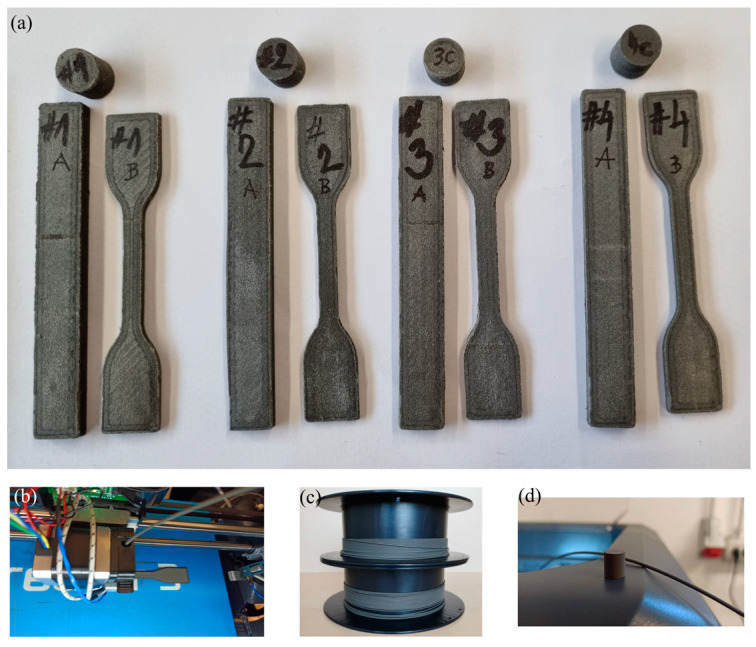
Images of FDM printed tensile, flexural test samples, and cylinders (**a**), FDM printing of tensile test sample (**b**), extruded and spooled filaments (**c**), and FDM printed cylindrical magnet (**d**).

**Figure 3:**
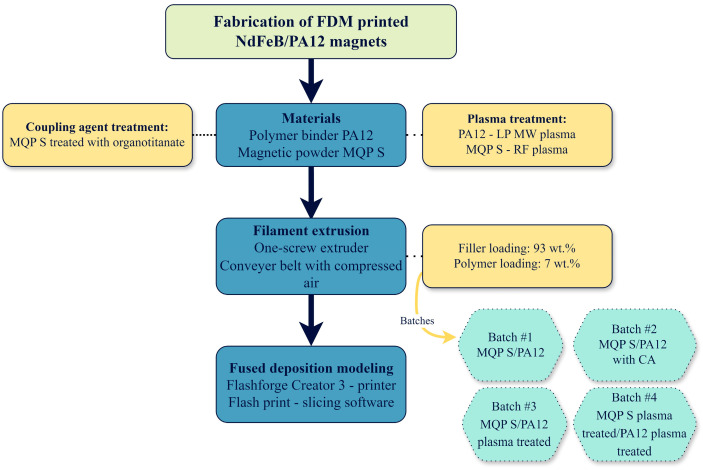
Process diagram highlighting the major steps from material selection to FDM printed parts.

The PBMs used in this study were fabricated using an industrial-grade desktop 3D printer, Flashforge Creator 3 (Jinhua, Zhejiang, China), accompanied by its slicing software, Flash Print version 5.5.2. Printing was performed at a temperature of 270 °C with a print speed of 50 mm/s, while the travel speed was adjusted to 100 mm/s. The printing platform was preheated to 80 °C to facilitate optimal adhesion of the extruded material. To further enhance bed adhesion and prevent detachment during the printing process, each specimen was printed with a 6-layer brim. Considering the risk of clogging during extrusion, a nozzle head with a 0.8 mm diameter was selected. The gap between the nozzle head and the print bed was set to 0.2 mm. The extruder was primed with a single skirt layer at the beginning of each print. The samples were printed in a horizontal orientation on the build platform, adopting a raster angle configuration of ±45° for alternate layers and ensuring an infill density of 100%. Cylindrical specimens measuring 10 mm × 9.50 mm were produced to evaluate the magnetic properties and environmental stability of the printed samples. For mechanical assessments, dog-bone-shaped samples with dimensions of length 75 mm, width 12.5 mm, thickness 4 mm, and gauge length 20 mm were printed to determine the tensile properties in accordance with the ISO 527-2 standard [40]. The samples for the flexural test had dimensions of 80 mm length, 10 mm width, and 4 mm thickness, in accordance with the ISO 178 standard [41]. A detailed summary of the printing parameters is presented in Table 2, while Figure 2 illustrates the spooled filaments and the printed samples, and Figure 3 outlines the process flow from material preparation to FDM printing.

### 2.5. Characterisation

Various techniques have been employed to characterize the physical, magnetic, and mechanical properties of filaments and FDM-printed samples derived from four distinct batches. A review of the literature on polyamide activation by plasma treatments indicates varied findings, with discussions on functional group types often avoided owing to the difficulties of overlapping peaks when using standard XPS for surface characterization [41,42,43]. However, wettability is determined by the straightforward technique, i.e., interaction with water. A clear distinction emerged when observing the behavior of polymer powders in water:plasma-treated PA12 dispersed effectively, indicating an improved affinity for water, whereas untreated PA12 remained floating on the water surface. Obviously, plasma treatment of polymer powder resulted in a significant increase in wettability. The quantitative technique of the sessile drop method is not feasible for fine powder because the water droplet is larger than the powder.

The surface morphology and elemental composition of filaments and fractured surfaces of mechanical test tubes after the tensile test were analyzed via scanning electron microscopy (SEM) coupled with energy-dispersive X-ray analysis (EDX). SEM micrographs and EDX spectra were acquired in the secondary electron mode using a Jeol–IT300 SEM equipped with an Oxford EDX system, operating at a beam energy of 15 kV. Notably, no pre-treatment was applied to the samples, which were fixed onto carbon tape prior to analysis.

The filaments from all four batches were subjected to thermogravimetric analysis (TGA) to assess their temperature stability and filler content. TGA tests were performed using a Mettler Toledo thermogravimetric analyzer TGA/DSC1 model (Greifensee, Switzerland). Uniform test conditions were maintained for all batches in dynamic mode, with the samples subjected to heating from ambient temperature to 600 °C at a heating rate of 10 °C/min in the presence of air as the purge gas.

Differential scanning calorimetry (DSC) was employed to evaluate the thermal stability, influence of additives and fillers, melting behavior, and cold crystallization of the filaments. The DSC analysis was conducted using a Mettler Toledo DSC1 STARe System (Greifensee, Switzerland). The first cooling and the second heating run were examined, with the samples undergoing heating from 25 °C to 480 °C at a rate of 10 °C/min under an air atmosphere to determine melting behavior. Additionally, a cooling cycle was performed between two heating cycles, involving a temperature range of 200–30 °C at a rate of 10 °C/min in an air atmosphere to ascertain cold crystallization. The degree of crystallinity (*X_c_* [%]) was calculated using the following formula:

*X*_c_ = Δ*H*_m_/((1 − *w*_f_) × Δ*H*_0_) × 100, (1)

where Δ*H*_m_ [J/g] represents the melting enthalpy after the second heating, *w_f_* is the weight fraction of the filler [%], and Δ*H*_0_ [J/g] corresponds to the melting enthalpy [J/g] of 100% crystalline PA12 (245 J/g [12,44]).

To assess the rheological properties of the filaments, melt flow index (MFI) measurements were conducted using an LMI5000 Series instrument (Dynisco, Franklin, MA, USA). The filaments were granulated and 30 g of each batch was introduced into an MFI capillary and preheated to 260 °C. The melt time was set at 120 s and a 5 kg load was applied.Archimedes’ principle was employed using an analytical balance (XP205 by Mettler-Toledo, Greifensee, Switzerland) to determine the densities of the printed cylinders. The measured densities were compared with the calculated densities, which were determined using the rule of mixtures, not accounting for the void presence, using the following equation:

*ρ*_calculated_ = (*ρ*_filler_ × *vol*%_filler_
*+ ρ*_add1_ × *vol*%_add1_
*+ ρ*_add2_ × *vol*%_add2_
*+ ρ*_add3_ × *vol*%_add3_
*+ ρ*_polymer_ × *vol*%_polymer_)/100, (2)

where labels add1, add2, and add3 refer to the following additives: the coupling agent, internal lubricant, and external lubricant, respectively.

Porosity was evaluated using the following equation:

*Porosity* (%) = (*ρ*_calculated_
*− ρ*_measured_)/*ρ*_calculated_ × 100, (3)

where *ρ*_calculated_ represents the calculated density and *ρ*_measured_ denotes the measured density.To investigate the internal structure and assess the porosity, potential voids, or interlayer air gaps, one sample from each batch was subjected to high-resolution X-ray computed tomography (CT) scanning. The scans were performed using a Phoenix v|tome|x s240 system (General Electric, Cincinnati, OH, USA). The operational parameters for the CT scans were set to a voltage of 170 kV and current of 230 µA, with an exposure time of 200 ms per projection. The acquired data were reconstructed to visualize and quantify the internal morphology of the printed samples, enabling the analysis of their structural integrity.The FDM-printed cylinders were subjected to magnetization using an impulse magnetizer K-Series (MAGNET-PHYSIK; Köln, Germany) at a voltage of 2000 V to reach saturation. Following magnetization, re sidual remanence (B_r_) and intrinsic coercivity (H_ci_) were determined using a permeameter (PERMAGRAPH^®^, MAGNET-PHYSIK, Köln, Germany). The measured residual remanence was compared to the theoretical value, which was calculated using the following formula:

*Br*_theorethical_ = *vol*%_filler_/100 × *Br*_as-received powder_, (4)

The magnetic flux of the magnetized cylinders was measured both before and after subjecting them to environmental tests using a Helmholtz coil (MS 75 with an electronic Fluxmeter EF 14, MAGNET-PHYSIK, Köln, Germany).

Mechanical testing was performed at room temperature on five samples from each batch. The mechanical evaluation included both flexural and tensile tests. Flexural tests were performed at a testing speed of 1 mm/min using a Zwick test machine (Z100, Zwick Roell, Ulm, Germany) in accordance with the ISO 178:2001 standard. Following the ISO 527-2 guidelines, tensile testing was also conducted using the same testing apparatus employed for flexural testing, maintaining a testing speed of 1 mm/min. A load cell with a capacity of 100 kN was used in the tests.

### 2.6. Evaluation of Environmental Stability and Corrosion Resistance

Two test scenarios were employed to assess the environmental stability and corrosion resistance of FDM-printed cylinders. The magnetic flux was measured both before and after each test, with a minimum of three samples. The first test aimed to evaluate magnet stability in a high-temperature aqueous environment, targeting the potential influence of water absorption on flux loss. The magnetized cylinders were immersed in deionized (DI) water at 85 °C for 1000 h. Additionally, a bulk corrosion test (BCT) was conducted to evaluate the degradation of the material under elevated temperature and exposure to water vapor. The cylinders were subjected for 500 h to pressurized steam at 120 °C and 2 bars to simulate conditions that may lead to accelerated material deterioration and, consequently, flux loss.

## 3. Results and Discussion

### 3.1. Characterisation of the Filaments

**Figure 4:**
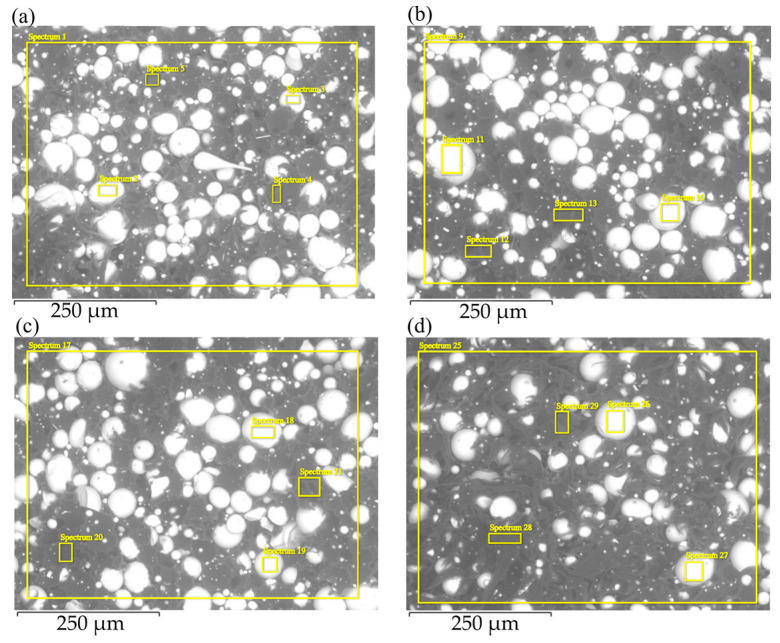
SEM micrographs of filament fractured surface for batches (**a**) MQP S/PA12, (**b**) MQP S/PA12 CA, (**c**) MQP S/PA12*, and (**d**) MQP S*/PA12*. Note that spherical lighter-colored particles represent MQP S particles, while the darker material corresponds to the PA12 matrix.

SEM micrographs of fractured surfaces of filaments for all four batches are shown in Figure 4. Table 3, Table 4, Table 5 and Table 6 present the elemental compositions (wt.%) of major elements detected on the cross-sectional areas of the filament surfaces by EDX, including both the magnetic powders and the polymer matrix within the filament. Examination of the SEM images revealed no noticeable contrast variation among batches. The homogeneity and distribution of the magnetic particles within the polymer matrix were consistent across all batches. The voids observed in the images suggest a debonding mechanism in which the MQP S particles are pulled from the matrix. The elemental compositions of all filament batches were comparable, with a rather high concentration of carbon (C) observed in the spectra MQP S. This high C concentration is likely attributed to the examination of cross-sections involving filament breakage by pulling. It appears that when the filament was torn apart, the MQP S particles retained some polymer on their surfaces, contributing to the high concentration of C observed in the spectra of MQP S.

**Figure 5:**
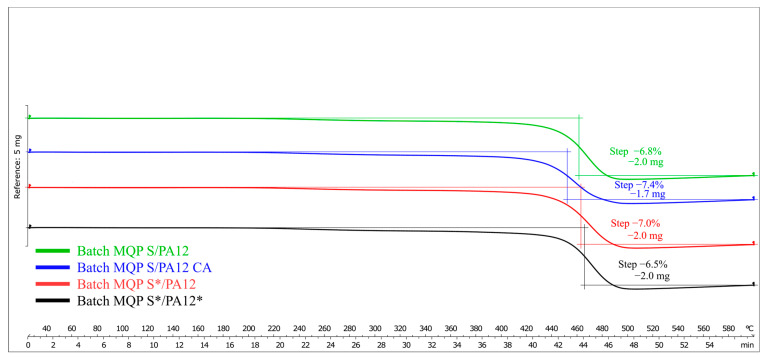
TGA curves depicting the thermal degradation behavior of filaments from all four produced batches.

The results obtained from the TGA analysis are shown in Figure 5. TGA offers a useful means of estimating polymer content, as the percentage of mass loss corresponds to the extent of polymer degradation. As expected, across all four batches, each containing 7 wt.% polymer, the mass loss remained close to 7%, as demonstrated in Figure 5. Although there were minor variations among the batches, these fluctuations suggest that the feedstock materials may not have been perfectly homogenized during filament production. Notably, the second batch (blue curve in Figure 5), which incorporated organo-titanate as a coupling agent, exhibited the highest mass loss due to the partial degradation of the coupling agent itself.

**Figure 6:**
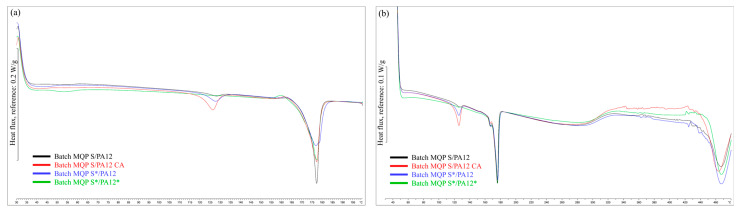
First (**a**) and second (**b**) heating DSC curves for extruded filaments.

Results of the DSC analysis, presented in Figure 6 and Table 7, revealed that the melting temperatures (T_m1_ and T_m2_) after the first and second heating cycles for all four batches were consistently close, with T_m1_ at approximately 176 °C and T_m2_ at approximately 175 °C. This uniformity suggests similar thermal stability and composition among batches in terms of the factors influencing the melting points. The enthalpy changes (ΔH_m1_ and ΔH_m2_) after the first and second heating cycles showed minor variations among batches, with values in the second heating cycle (ΔH_m2_) being slightly lower, possibly owing to decreased crystallinity or structural changes [44]. The calculated degree of crystallinity ranged from 19.5% to 21.7%. Notably, the batch MQP S/PA12 CA exhibited slightly higher crystallinity, suggesting that the presence of organo-titanate as a coupling agent contributed to the increased crystalline structure formation. Similarly, in the case of MQP S*/PA12*, where both the filler and binder underwent plasma surface modification, the higher degree of crystallinity can be attributed to these modifications.

The MFI data for all batches of filament are presented in Table 8. The batch labeled MQP S/PA12 CA exhibited the highest MFI among all the batches. A higher MFI can be attributed to the presence of a coupling agent in the form of organo-titanate, which enhances the dispersion between the inorganic filler phase and organic polymer phase. Consequently, it promotes effective deagglomeration and the subsequent removal of air and water from the interface [45]. Conversely, the batch in which only the polymer underwent treatment in a low-pressure microwave plasma reactor displayed the lowest MFI. Similar plasma treatment resulted in suboptimal polymer melting behavior, particularly when prolonged low-pressure microwave plasma treatment was applied to achieve target morphological changes [33]. These changes proved to be more advantageous when the magnetic filler was also subjected to radio-frequency plasma treatment, as observed in the case of batch MQP S*/PA12*. Importantly, suboptimal MFI values can lead to challenges in the extrusion process, such as over-extrusion or inconsistent filament extrusion, impacting the quality and consistency of FDM-printed samples.

### 3.2. Properties of the FDM-Printed Polymer-Bonded Magnets

Table 9 provides an overview of the measured and calculated densities of FDM-printed cylinders, along with the corresponding calculated porosity. It is noteworthy that in a prior study, injection-molded cylinders produced from the same feedstock materials as batch MQP S/PA12 CA achieved a measured density exceeding 4.5 g/cm^3^ and exhibited a calculated porosity of less than 2%. In the context of this study, the achieved FDM densities ranged from 92% to 94% of the theoretical density, indicating a reasonably high level of material consolidation. Nevertheless, it is important to acknowledge that FDM-printed cylinders inherently possess higher porosity compared to injection-molded ones, due to the nature of the printing process itself. FDM technology is known to yield parts with increased porosity when compared to those produced through alternative manufacturing methods [46]. Given that identical printing parameters were applied across all batches, the observed higher porosity in the FDM cylinders can be attributed to potential challenges in achieving optimal layer-to-layer adhesion during the printing process.

**Figure 7:**
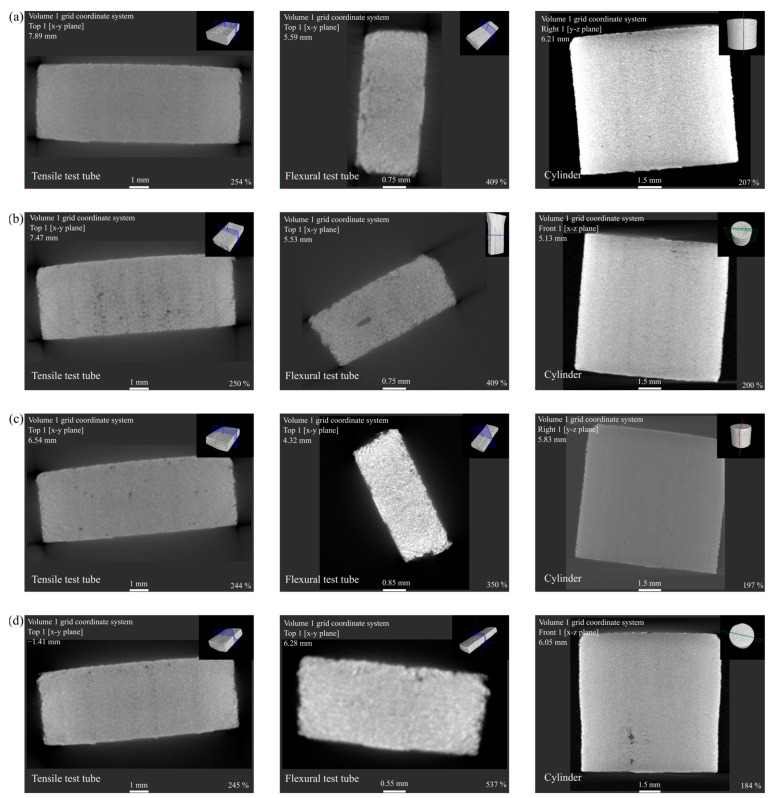
CT scan comparison of FDM-printed samples in batches: (**a**) MQP S/PA12, (**b**) MQP S/PA12 CA, (**c**) MQP S/PA12*, and (**d**) MQP S*/PA12*.

The CT scan analysis across all batches, including tensile test tubes, flexural test tubes, and cylinders, showed that the cylinders exhibited the least detectable voids. This suggests a correlation between the smaller dimensional scale of the cylinders and a reduction in observable porosity, potentially due to the layer resolution and deposition characteristics inherent to FDM. In contrast, mechanical test tubes manifested a greater frequency of air voids, indicating a variance in the filament-to-filament adhesion during the printing process. In the reference batch (MQP S/PA12), voids are present sporadically, which could be indicative of a uniform filler distribution that is not influenced by a coupling agent. Contrastingly, introducing an organo-titanate coupling agent in batch MQP S/PA12 CA is associated with the most detectable voids and air gaps. This observation is particularly interesting when considering the batch’s highest MFI value. The elevated MFI could imply a reduced viscosity, potentially facilitating the printing process but also allowing for increased void formation, likely due to the material flowing too readily, leading to inconsistent layer adhesion and increased porosity. Similarly, batch MQP S/PA12* also exhibited a significant presence of voids. The lowest MFI value of this batch suggests that the increased viscosity could have adversely affected the printing process, possibly hindering the material’s ability to flow and merge between layers, thus contributing to the formation of voids [47]. The batch MQP S*/PA12* voids may signal an altered interaction between the modified surfaces of the filler and the polymer, potentially influenced by the interaction between the altered surface-free energies of plasma-treated filler and polymer [33]. Detailed visualisation of these characteristics can be seen in the CT scan depicted in Figure 7.

When it comes to magnetic properties and residual remanence (B_r_) in PBMs, they are significantly influenced by the volume fraction of the magnetic filler within the magnet. Generally, higher volume percentages of filler lead to increased B_r_ values. However, the presence of pores can have a counteractive effect, diminishing the achieved B_r_ [48]. In Table 10, both measured and theoretical B_r_ values for FDM-printed cylinders are presented. Across all batches, the achieved Br values ranged from 85% to 89% of their theoretical counterparts. Notably, a correlation can be observed between measured porosity values from Table 9 and the achieved B_r_ values; samples with higher porosity tend to exhibit lower B_r_ values. Specifically, batches MQP S/PA12 CA and MQP S/PA12* had porosity levels of 8.4% and 8%, respectively, achieving B_r_ values of 85% and 86% of their theoretical B_r_. Conversely, batches MQP S/PA12 and MQP S*/PA12*, which had lower porosity at approximately 6%, achieved nearly 89% of their theoretical B_r_ values.

In Table 10, measured values for intrinsic coercivity (H_ci_) can be observed. Considering that the as-received MQP S powder exhibits an H_ci_ range of 670–750 kA/m [35], it becomes evident that certain FDM batches displayed H_ci_ lower than that of the as-received MQP S powder. This decrease in H_ci_ can be attributed to inappropriate processing conditions that may have altered the microstructure of the materials. Poor adhesion between the polymer matrix and the magnetic particles results in a reduction in coercivity. It is worth mentioning that during filament extrusion, the feedstock material, including MQP S powder, was exposed to a temperature range of 180 °C to 200 °C for a 30-min duration. This thermal exposure likely contributed to the oxidation of MQP S particles. Furthermore, any occurrence of larger grain sizes or misaligned grains during the printing process could lead to a decrease in H_ci_ values. Interestingly, the batch containing a coupling agent stands out as the exception, maintaining the same H_ci_ values as the as-received MQP S powder. This further confirms that the coupling agent could effectively shield the magnetic powder during filament manufacturing and extrusion processes.

Tensile test results of batches of FDM-produced composites, presented in Table 11, provide an overview of how different treatments and the inherent characteristics of FDM, such as increased porosity and layer adhesion variability, impact the mechanical properties of these materials. All batches exhibited variations in elastic modulus as well as relatively large standard deviations, indicating potential inconsistencies in material properties. This variability could stem from the FDM process itself, where issues in layer consolidation can lead to poor interlayer adhesion, reducing the structural integrity of the printed parts. Additionally, the presence of porosity within the layers is a common concern in FDM printing, further contributing to potential weaknesses in the mechanical structure and impacting the elastic modulus measurements [49]. The addition of 0.5 wt.% of organo-titanate in MQP S/PA12 CA led to a reduced modulus of 141 ± 40 MPa when compared to MQP S/PA12, indicating enhanced flexibility but at the cost of increased brittleness, as evidenced by its significantly lower ductility (5 ± 2%). In the third batch, MQP S/PA12*, where PA12 was treated with low-pressure microwave plasma, a considerable increase in stiffness was observed, with an elastic modulus of 974 ± 161 MPa. This suggests that plasma treatment improves material properties, likely due to enhanced adhesion at the molecular level. The final batch, MQP S*/PA12*, which underwent dual plasma treatment, presented a balanced profile of moderate stiffness (578 ± 389 MPa) and the highest ductility (21 ± 2%), indicating an optimal combination of treatments for improved flexibility and consistency. Across all batches, tensile strength values were relatively similar, ranging from 5.5 to 6.9 MPa. Despite the different treatments, this uniformity suggests that while FDM-produced materials can withstand some tension, their strength in resisting a pulling force is not exceptionally high, which is a characteristic to be considered in their application. Overall, the results show that the tensile properties of FDM-produced materials, particularly elastic modulus and elongation at break, vary significantly across different batches, emphasizing the crucial role of specific treatments in material optimization. Evidently, microwave and radiofrequency plasma treatments have been shown to enhance these properties markedly. Despite these variations, the tensile strength across all batches remains consistently limited, underscoring a characteristic trait of FDM materials in their response to tensile stress.

When it comes to flexural properties, presented in Table 12, the first batch, MQP S/PA12, which combined MQP S filler with PA12 without coupling agents, demonstrated a flexural strength of 14 ± 2 MPa and a strain of 6 ± 2%. This serves as a reference for the untreated material’s performance. In contrast, the second batch, MQP S/PA12 CA, which included an organo-titanate coupling agent, showed a lower flexural strength of 10 ± 1 MPa but a slightly higher strain of 7 ± 1%, suggesting that the addition of the coupling agent may lead to a decrease in strength but a marginal increase in flexibility. The third batch, MQP S/PA12*, where PA12 underwent microwave plasma treatment, exhibited a flexural strength of 10 ± 1 MPa and an increased strain of 8.6 ± 0.3%, indicating that plasma treatment of the polymer powder can enhance the material’s flexibility. This is evident in the higher strain value compared to the untreated and coupling agent-treated batches. The final batch, MQP S*/PA12*, where both MQP S and PA12 underwent radiofrequency and microwave plasma treatment, respectively, showed the highest flexural strength of 15.4 ± 0.4 MPa and a strain of 5 ± 1%. This suggests that the dual plasma treatment significantly enhances the material’s strength while maintaining a comparable level of flexibility to the untreated batch. These results highlight the influence of material treatments on the flexural properties of FDM-printed composites. The inclusion of a coupling agent and plasma treatments, particularly dual plasma treatment, has been found to significantly alter the flexural strength and strain, demonstrating the potential for tailoring material properties through specific processing techniques in FDM applications.

**Figure 8:**
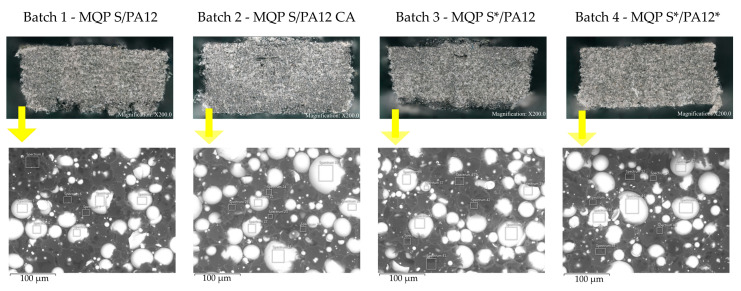
SEM images of the fractured surfaces after the tensile test. Note that spherical lighter-colored particles represent MQP S particles, while the darker material corresponds to the PA12 matrix.

Figure 8 displays SEM micrographs of the fractured surfaces from each batch after the tensile test. It is evident from the images that the breakage occurs directly along the magnet/polymer interface, suggesting that delamination is more likely to occur in regions with smooth uniform surfaces compared to areas with irregularly shaped particles [50]. This observation underscores the importance of particle morphology in influencing the adhesive bonding within the composite, potentially affecting the overall mechanical integrity of the printed structures.

### 3.3. Evaluation of Environmental Stability and Corrosion Resistance

Environmental stability assessment of the FDM-printed magnets involved subjecting them to two distinct test scenarios. Magnetic flux measurements were taken before and after each test and the results are presented in Table 13 and Table 14, distinguishing between reversible and irreversible flux losses. When the magnets were immersed in deionized water at 85 °C for 1000 h, both reversible and irreversible flux losses were remarkably low, with the irreversible loss being below the detection limit. This finding suggests that despite the inherently higher porosity of the FDM-printed samples compared to magnets produced using alternative formative manufacturing technologies, the former can find industrial applications in environments where exposure to water solutions at elevated temperatures is expected. Similarly, Table 14 illustrates the flux loss after the BCT. In this case, as well, the magnets exhibited minimal flux loss, indicating their resistance to hygrothermal corrosion. The consistently low or zero irreversible flux loss observed after both tests underscores the potential of FDM technology in rapid prototyping. Additionally, minimal surface rust was observed at the conclusion of both tests, affirming the material’s durability.

## 4. Conclusions

This research has demonstrated the effectiveness of FDM technology in fabricating Nd–Fe–B magnets bonded with polyamide 12, utilizing plasma treatments to improve the adhesion between inorganic magnetic and organic polymer powder. The utilization of low-pressure radio frequency plasma for Nd–Fe–B and low-pressure microwave plasma for PA12 has enhanced the mechanical strength of FDM-printed magnets without compromising their magnetic performance. Key findings include the achievement of material densities ranging from 92% to 94% of theoretical values, with magnetic remanence across batches reaching 85% to 89% of theoretical predictions. Tensile strength values were consistently reported between 5.5 to 6.9 MPa across all treatments, highlighting the uniform resilience of the materials under tension. Flexural testing further illustrated the impact of processing techniques on material properties. The dual plasma-treated batch showcased the highest flexural strength at 15.4 ± 0.4 MPa, with a strain of 5.3 ± 0.5%, indicating a significant enhancement in material strength while maintaining rather adequate flexibility.

Environmental stability assessments underscored the materials’ resistance to hygrothermal corrosion, with both reversible and irreversible flux losses remaining remarkably low or negligible (below the detection limit) after 1000 h of immersion in deionized water at 85 °C. Bulk corrosion tests supported these findings, demonstrating minimal flux loss and indicating superior durability and resistance of the plasma-treated magnets to environmental stressors.

The integration of plasma treatments with FDM printing technology offers a promising pathway to produce Nd–Fe–B/PA12 polymer-bonded magnets, achieving advancements in mechanical strength and magnetic properties, without deterioration of environmental stability. This study underlines the potential of specific plasma treatments in optimizing the performance of FDM-printed magnetic materials, paving the way for their application in a variety of demanding environments.

Despite these promising results, there are some limitations to consider. Firstly, the FDM process results in materials with lower density and increased porosity compared to conventional technologies like injection molding. This may limit the suitability of FDM for applications requiring fully dense materials. Additionally, the manufacturing of printable filament remains challenging and may require further optimization for large-scale applications. While this study has demonstrated significant advancements in mechanical strength and magnetic properties, further research is needed to address these limitations and explore scalable and versatile manufacturing solutions for advanced magnetic applications.

## Figures and Tables

**Table 1:** Overview of manufactured filament batches.

Batch Label	Magnetic Filler	Polymer Binder	Coupling Agent	Surface Treatment
MQP S/PA12	Nd–Fe–B	Polyamide 12	None	None
MQP S/PA12 CA	Nd–Fe–B	Polyamide 12	Organo-titanate	None
MQP S/PA12*	Nd–Fe–B	Polyamide 12	None	Low-pressure microwave (MW) plasma *
MQP S*/PA12*	Nd–Fe–B	Polyamide 12	None	Radiofrequency plasma * and low-pressure microwave plasma *

* indicates plasma treatment.

**Table 2:** Detailed printing parameters for the FDM fabrication.

Printing Parameter	Value [Unit]
Material	MQP S bonded with PA12
Nozzle temperature	270 °C
Printing speed	50 mm/s
Travel speed	100 mm/s
Platform temperature	80 °C
Nozzle head diameter	0.8 mm
Raster angle	±45°
Infill density	100%
Infill pattern	Hexagon
Layer thicknesses	0.15 mm

**Table 3:** Elemental compositions (wt.%) detected in the cross-sectional areas of the filament surface of batch MQP S/PA12.

	MQP S		PA12	
	Sp. 2	Sp. 3	Sp. 4	Sp. 5
C	48.4	49.5	72.3	74.3
O	4.9	4.8	9.2	9.0
Ti	1.1	1.1	0.4	0.4
Fe	33.1	33.2	13.4	12.0
Co	1.7	1.3	0.5	0.5
Zr	2.2	2.1	0.8	0.7
Nd	8.6	8.0	3.4	3.1
Total	100.0	100.0	100.0	100.0

**Table 4:** Elemental compositions (wt.%) detected in the cross-sectional areas of the filament surface of batch MQP S/PA12 CA.

	MQP S		PA12	
	Sp. 10	Sp. 11	Sp. 12	Sp. 13
C	44.5	46.4	70.6	72.3
O	4.9	5.5	9.4	9.7
Ti	1.1	1.0	0.6	0.5
Fe	36.6	34.6	14.1	12.9
Co	1.7	1.5	0.7	0.5
Zr	2.1	2.0	0.9	0.7
Nd	9.1	9.0	3.7	3.4
Total	100.0	100.0	100.0	100.0

**Table 5:** Elemental compositions (wt.%) detected in the cross-sectional areas of the filament surface of batch MQP S/PA12*.

	MQP S		PA12	
	Sp. 18	Sp. 19	Sp. 20	Sp. 21
C	47.0	46.2	71.5	71.2
O	4.4	4.3	9.1	7.7
Ti	1.1	1.1	0.4	0.4
Fe	35.3	35.7	13.7	15.2
Co	1.4	1.3	0.6	0.6
Zr	2.0	2.2	0.9	0.9
Nd	8.8	9.2	3.8	4.0
Total	100.0	100.0	100.0	100.0

**Table 6:** Elemental compositions (wt.%) detected in the cross-sectional areas of the filament surface of batch MQP S*/PA12*.

	MQP S		PA12	
	Sp. 26	Sp. 27	Sp. 28	Sp. 29
C	50.6	51.3	76.5	74.3
O	4.8	4.7	10.8	9.5
Ti	1.1	0.9	0.3	0.3
Fe	32.2	31.8	9.1	11.5
Co	1.3	1.3	0.4	0.4
Zr	1.8	1.8	0.6	0.8
Nd	8.2	8.2	2.3	3.2
Total	100.0	100.0	100.0	100.0

**Table 7:** Overview of DSC analysis for filament batches, including melting temperatures (T_m1_ and T_m2_), enthalpy (ΔH_m1_ and ΔH_m2_) after first and second heating cycles, cold crystallization temperature (T_c_), enthalpy (ΔH_c_) after the cooling cycle, and calculated degree of crystallinity (W_c_).

Batch	T_m1_ (°C)	ΔH_m1_ (J/g)	T_c_ (°C)	ΔH_c_ (J/g)	T_m2_ (°C)	ΔH_m_ (J/g)	W_c_ (%)
MQP S/PA12	176.8	4.4	150.0	3.9	175.4	3.4	19.5
MQP S/PA12 CA	176.7	4.3	148.2	4.2	175.4	3.7	21.7
MQP S/PA12*	176.4	4.1	149.6	3.9	175.7	3.6	20.8
MQP S*/PA12*	176.5	4.3	150.0	4.0	175.4	3.7	21.4

**Table 8:** Summary of the MFI data for each batch.

Batch	MFI [g/10 min]	Standard Deviation
MQP S/PA12	216	21
MQP S/PA12 CA	289	9
MQP S/PA12*	185	12
MQP S*/PA12*	239	10

**Table 9:** Measured and theoretical densities along with porosity of FDM-printed cylinders for each batch.

Batch	Measured Density	Vol% Filler *	Theoretical Density	Porosity [%]
MQP S/PA12	4.5 ± 0.1	65.5	4.8	6.0 ± 0.4
MQP S/PA12 CA	4.4 ± 0.1	66.9	4.8	8.4 ± 1.1
MQP S/PA12*	4.4 ± 0.1	65.5	4.8	8.0 ± 0.5
MQP S*/PA12*	4.5 ± 0.1	65.5	4.8	5.9 ± 0.7

* including additives and excluding void formation.

**Table 10:** Summary of magnetic properties for FDM printed cylinders: maximum energy product (B H_max_), measured and theoretical remanence (B_r_), and intrinsic coercivity (H_ci_).

Batch	BH_max_ [kJ/m^3^]	H_ci_ [kA/m]	Br_measured_ [mT]	Br_theoretical_ [mT]	Br_measured_ [% of Br_theoretical_]
MQP S/PA12	29.4 ± 0.4	650 ± 1	432.3 ± 3.5	488.1	88.6
MQP S/PA12 CA	28.3 ± 0.1	710.2 ± 0.8	422.1 ± 0.8	498.7	84.7
MQP S/PA12*	28.0 ± 0.4	649.7 ± 1.2	422.1 ± 2.7	488.1	86.5
MQP S*/PA12*	27.9 ± 1.5	650	434.4 ± 0.8	488.1	89.0

**Table 11:** Overview of elastic modulus, tensile strength, and elongation at break for different batches of 7 wt.% for PA12 and 93 wt.% for Nd–Fe–B FDM-produced composites.

Batch	Elastic Modulus [MPa]	Tensile Strength at Yield [MPa]	Elongation at Yield [%]
MQP S/PA12	621 ± 247	6.9 ± 0.3	17 ± 4
MQP S/PA12 CA	141 ± 40	6 ± 1	5 ± 2
MQP S/PA12*	974 ± 161	5.5 ± 0.3	16 ± 4
MQP S*/PA12*	578 ± 389	6 ± 1	21 ± 2

**Table 12:** Overview of flexural strength and strain for different batches of 7 wt.% PA12 and 93 wt.% Nd–Fe–B FDM-produced composites.

Batch	Flexural Strength [MPa]	Flexural Strain [%]
MQP S/PA12	14 ± 2	6 ± 2
MQP S/PA12 CA	10 ± 1	7 ± 1
MQP S/PA12*	10 ± 1	8.6 ± 0.3
MQP S*/PA12*	15.4 ± 0.4	5 ± 1

**Table 13:** Results of the tests performed by immersion in water at 85 °C for 1000 h show reversible and irreversible flux losses (%).

Batch	Reversible Flux Loss [%]	Irreversible Flux Loss [%]	Rusting
MQP S/PA12	1	0	Low
MQP S/PA12 CA	0.6	0	Low
MQP S/PA12*	0.6	0	Low
MQP S*/PA12*	1.4	0	Low

**Table 14:** Bulk corrosion test results at 120 °C for 500 h show reversible and irreversible flux losses (%).

Batch	Reversible Flux Loss [%]	Irreversible Flux Loss [%]	Rusting
MQP S/PA12	2.8	0.5	Low
MQP S/PA12 CA	3.6	0.4	Low
MQP S/PA12*	2.5	0.1	Low
MQP S*/PA12*	2.3	0.4	Low

## Data Availability

Data are contained within the article.
